# Polymorphisms of the *BMPR1B*, *BMP15* and *GDF9* fecundity genes in four Chinese sheep breeds

**DOI:** 10.5194/aab-67-51-2024

**Published:** 2024-02-07

**Authors:** Jinxin Wang, Yufang Liu, Siwu Guo, Ran Di, Xiangyu Wang, Xiaoyun He, Mingxing Chu

**Affiliations:** 1 State Key Laboratory of Animal Biotech Breeding, Institute of Animal Science, Chinese Academy of Agricultural Sciences (CAAS), Beijing 100193, China

## Abstract

Numerous studies on prolific sheep breeds have shown that the transforming growth factor beta (TGF- β ) superfamily members, including bone morphogenetic protein receptor type 1B (*BMPR1B*), bone morphogenetic protein 15 (*BMP15*) and growth differentiation factor 9 (*GDF9*), are the essential regulators of ovulation rate and litter size. In total, 11 known mutations (1 in *BMPR1B*, 6 in *BMP15* and 4 in *GDF9*) are able to increase the ovulation rate and litter size, respectively. In this study, the genomic DNA was isolated from 512 high-prolificacy sheep (including the Small-tailed Han, Hu and Wadi sheep breeds) and 164 low-prolificacy sheep (Tan sheep), and genotyping of the specific mutations of the three fecundity-related genes was carried out by sequencing. The results showed that the *FecB* mutation in *BMPR1B* was detected in all four sheep breeds, and the frequency of B allele was significantly higher in the high-prolificacy breeds than that in the low-prolificacy breed ( P<0.001 ). A novel mutation, c.T755C (named S1), was found in *BMP15* from the four sheep breeds. However, known mutations such as *FecX* ${}^{I}$ , *FecX* ${}^{H}$ , *FecX* ${}^{B}$ , *FecX* ${}^{G}$ , *FecX* ${}^{L}$ and *FecX* ${}^{R}$ were not detected in these breeds. Three known loci (G1, G3 and G4) and a new mutation, c.A1515G (named S2), were found in *GDF9*, and the other three known mutations (*FecG* ${}^{H}$ (G8), *FecG* ${}^{E}$ and *FecTT*) were not detected in all four sheep breeds. The genotype distribution at the G1 and G4 loci had significant differences between the low-prolificacy sheep breed and the other three high-prolificacy sheep breeds. There was no difference in the genotype distribution at the G1 and G4 loci between the three high-prolificacy sheep breeds. Haplotype analysis of the four polymorphic loci in *GDF9* suggested that H4 (GGAA) was the preponderant haplotype in the three high-prolificacy sheep breeds, but H1 (GGGG) was in the low-prolificacy sheep breed. These results preliminarily showed that the *BMPR1B* and *GDF9* might be major genes influencing the prolificacy of Chinese sheep breeds.

## 1 Implications

Many studies have shown that the reproductive performance of sheep is affected by genetic variation. Finding polymorphism of genes can provide potential molecular genetic markers for sheep reproduction. This study investigated the polymorphisms of three major reproductive genes (*BMPR1B*, *BMP15* and *GDF9*) in Chinese sheep and found that *BMPR1B* and *GDF9* might be the major genes influencing the reproductive performance of Chinese sheep breeds. This result might contribute to improving the prolificacy traits of Chinese native sheep in the future.

## 2 Introduction

Ovulation rate and litter size are important reproduction traits in sheep and are of high economic value. Reproduction traits typically have low to medium heritability and do not exhibit a noticeable response to phenotypic selection. Therefore, inclusion of genetic information of the genes associated with reproductive ability could efficiently enhance the selection response (Abdoli et al., 2016). In sheep, genetic variation in litter size and ovulation rate has been widely documented, and many findings have indicated that substantial differences between breeds and within breeds occurred in different mechanisms. Three members of the transforming growth factor- β (TGF- β ) superfamily, i.e., bone morphogenetic protein receptor type 1B (*BMPR1B*), bone morphogenetic protein 15 (*BMP15*) and growth differentiation factor 9 (*GDF9*), have been shown to be essential regulators for ovulation rate and follicular development (Davis, 2005). *GDF9* and *BMP15* are co-expressed exclusively in oocytes throughout most of the folliculogenesis and play central roles in controlling ovarian physiology (Stocker et al., 2020).

The *BMPR1B* known as *FecB* on sheep chromosome 6 includes 16 exons (gene ID 443454). The A–G transition was detected at nucleotide 746 of *BMPR1B* cDNA, causing a nonsynonymous substitution of glutamine with an arginine (Q249R) at position 249 of the mature protein in Booroola Merino sheep (Mulsant et al., 2001; Souza et al., 2001; Wilson et al., 2001). The *FecB* ${}^{B}$ allele leads to an additive effect for the ovulation rate and an increased litter size. The genotypes of *FecB* mutation in the ewes have been classified as homozygous carriers (BB) with more than five ovulations per estrous cycle, heterozygous carriers (B + ) with three to four ovulations and homozygous non-carriers ( ++ ) with an ovulation rate of two or less (Davis et al., 1982). A recent study of Small-tailed Han sheep showed that *BMPR1B* was highly expressed in hypothalamus, ovary, uterus and oviduct tissue during the follicular phase, and *BMPR1B* was expressed significantly more in the hypothalamus of polytocous ewes than in monotocous ewes during both the follicular and luteal phases (Wen et al., 2021). The *BMP15* known as *FecX* is on sheep chromosome X, and its coding sequence contains two exons (Galloway et al., 2000). Eight mutations have been detected within the sheep *BMP15*, including *FecX* ${}^{I}$ (Inverdale, V299D), *FecX* ${}^{H}$ (Hanna, Q291Ter), *FecX* ${}^{B}$ (Belclare, S367I), *FecX* ${}^{G}$ (Galway, Q239Ter), *FecX* ${}^{L}$ (Lacaune, C321Y), *FecX* ${}^{R}$ (Rasa Aragonesa, 154-159del WVQKSP), *FecXO* and *FecXGr* (Galloway et al., 2000; Hanrahan et al., 2004; Bodin et al., 2007; Martinez-Royo et al., 2008; Monteagudo et al., 2009; Abdoli et al., 2016; Demars et al., 2013). There are 13 identified variations in the Chinese Luzhong mutton sheep breed, of which 6 are novel (Di et al., 2021). All these mutations led to increased ovulation rates in the heterozygous animals and sterility in the homozygous animals. As a close homolog of *BMP15*, the mutations of *GDF9* also result in similar genotypes to those in *BMP15*, and they cause an increased ovulation rate in heterozygous ewes, while homozygotes are sterile (Hanrahan et al., 2004; McNatty et al., 2005a). The *GDF9* known as *FecG* is on sheep chromosome 5 and contains two exons. Eight single-nucleotide polymorphisms (SNPs), labeled G1–G8, have been identified in the entire coding region of the sheep *GDF9* (Hanrahan et al., 2004). Four mutations in *GDF9* have been identified as causing either infertility or an increased ovulation rate, including G8 (*FecG* ${}^{H}$ ) (Belclare and Cambridge, S395F) (Hanrahan et al., 2004), *FecG* ${}^{E}$ (Santa Ines, F345C) (Melo et al., 2008; Silva et al., 2011) and *FecTT* (Thoka, S427R) (Nicol et al., 2009). Two novel SNPs of *GDF9* (g.41768501A > G and g.41768485 G > A) were found in Luzhong mutton sheep and may be potential genetic markers for improving litter size (Wang et al., 2021).

Small-tailed Han sheep, Hu sheep and Wadi sheep are excellent native breeds in China for their significant characteristics of year-round estrus and hyper-prolificacy. Tan sheep is a native breed in China with seasonal estrus and single birth. The mean litter sizes of Small-tailed Han sheep, Hu sheep, Wadi sheep and Tan sheep have been reported to be 2.67, 2.77, 2.43 and 1.02 (China National Commission of Animal Genetic Resources, 2011), respectively. Based on the essential role of *BMPR1B*, *BMP15* and *GDF9* in the regulation of the ovulation rate and terminal folliculogenesis, three fecundity-related genes are considered possible candidate genes for prolificacy traits of sheep. Therefore, the present study was designed with the objectives of polymorphic study of three fecundity-related genes in high-prolificacy breeds (Small-tailed Han sheep, Hu sheep and Wadi sheep) and a low-prolificacy breed (Tan sheep) by direct sequencing after polymerase chain reaction (PCR) amplification.

## 3 Materials and methods

### 3.1 Animals and genomic DNA isolation

Blood samples (10 mL, jugular vein, ACD anticoagulant) were collected from 180 Small-tailed Han ewes (Jiaxiang Sheep Breeding Farm, Jiaxiang County, Shandong Province, China), 164 Tan ewes (Tan Sheep Breeding Farm, Yanchi County, Ningxia Hui Autonomous Region, China), 160 Wadi ewes (Zhanhua County, Shandong Province, China) and 172 Hu ewes (Yuhang Hu Sheep Breeding Farm, Hangzhou, Zhejiang Province, China). Genomic DNA was extracted from blood using the phenol-chloroform method, dissolved in TE buffer (10 mmol L ${}^{-1}$ Tris-HCl [pH 8.0], 1 mmol L ${}^{-1}$ EDTA [pH 8.0]) and kept at -20 ${}^{\circ }$ .

### 3.2 DNA sequencing

According to the DNA sequences of sheep (*Ovis aries*) (*BMPR1B*, NC_056059.1; *BMP15*, NC_056080.1; *GDF9*, NC_056058.1) and mRNA sequences of sheep (*Ovis aries*) (*BMPR1B*, XM_060416665.1; *BMP15*, NM_001114767.2; *GDF9*, NM_001142888.2), a total of five pairs of PCR primers were designed using Oligo 6.0 to amplify the sixth exon of *BMPR1B* (BMPR1B-6), the second exon of *BMP15* (BMP15-2) and the first (GDF9-1) and second (GDF9-2-1, GDF9-2-2) exons of *GDF9*. The primers were synthesized by Shenzhen BGI Co. Ltd. (Shenzhen, China). The primers, predicted product sizes and annealing temperatures are listed in Table 1.

**Table 1:** Primer sequence, product size and annealing temperature.

Primer name	Primers (5 ${}^{\prime }$ to 3 ${}^{\prime }$ )	Product size	Annealing
		(bp)	temperature ( ${}^{\circ }$ )
GDF9-1	F: GGATGAATAGGGTGTTGTC R: CTAACCTCCAGCAGCACTC	540	54
GDF9-2-1	F: TTAATTCACCACCAAAGCTATT R: GGGAAAAAGAAACTGTTTGAAG	878	55
GDF9-2-2	F: GTGTAAGATTGTCCCGTCA R: ATATAGCCTGCCAGGTTTC	1139	55
BMP15-2	F: ATTCATTTCAGGGCTGCTTG R: CAATGCTGAAGGCAAGGAAT	1104	55
BMPR1B-6	F: CTTTACCGCTACTGCCACCT R: CATGACATTCTTGGGCTCAG	499	54

PCR was carried out in a 20 µ L volume containing 0.15 µ mol L ${}^{-1}$ primer, 1× PCR buffer (50 mmol L ${}^{-1}$ KCl, 10 mmol L ${}^{-1}$ Tris-HCl [pH 8.0], 0.1 % Triton X-100), 2.0 mmol L ${}^{-1}$ MgCl ${}_{2}$ , 0.2 mmol L ${}^{-1}$ dNTP, 100 ng ovine genomic DNA and 0.05 U µ L ${}^{-1}$ Taq DNA polymerase (Promega, Madison, WI, USA), and the rest was ddH ${}_{2}$ O. The amplification conditions were as follows: initial denaturation at 94 ${}^{\circ }$ for 5 min, followed by 35 cycles of denaturation at 94 ${}^{\circ }$ for 30 s, annealing at 54–55 ${}^{\circ }$ for 45 s, extension at 72 ${}^{\circ }$ for 35 s, and a final extension at 72 ${}^{\circ }$ for 10 min on Mastercycler^®^ 5333 (Eppendorf AG, Hamburg, Germany). The PCR products were directly sequenced using the ABI3730 automatic sequencer (Perkin Elmer Applied Biosystems, Foster City, CA, USA) by Shenzhen BGI Co. Ltd. (Shenzhen, China).

### 3.3 Statistical analysis

Genotype distributions were analyzed using a chi-squared test. Linkage disequilibrium and haplotype analyses were performed using the SHEsis (Shi and He, 2005; Li et al., 2009) and Phase 2.1.1 (Stephens et al., 2001; Stephens and Donnelly, 2003) software.

## 4 Results

In the study, six SNPs were identified in exons of three fertility-related genes (*BMPR1B*, *BMP15* and *GDF9*) based on DNA sequence genotyping. Two new mutations, c.T755C (S1) in *BMP15* and c.A1515G (S2) in *GDF9*, were identified by analyzing four sheep breeds along with two other known mutations in *GDF9* (c.G477A, G3, and c.G721A, G4), and only c.A746G (*FecB*) in *BMPR1B* and c.T755C (G1) in *BMP15* were detected, in agreement with the 11 mutations in the previous study.

### 4.1 Genotyping of the *BMPR1B* mutation

**Figure 1:**
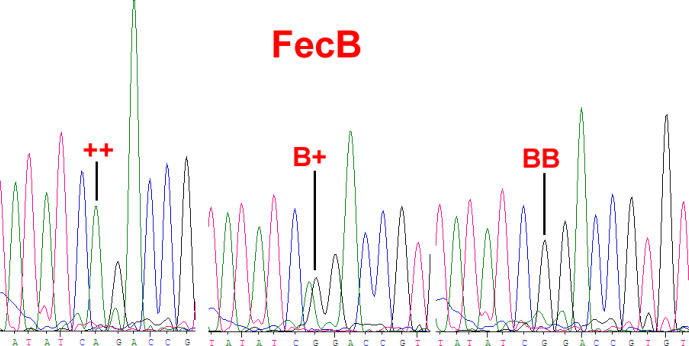
Sequence of genotypes ++ , B + and BB at the 746 loci in the Coding Sequence (CDS) region of *BMPR1B* sheep.

*BMPR1B* had two alleles: the “B” represented the *FecB* mutant nucleotide (carrier), and the “ + ” represented the wild-type nucleotide (non-carrier) (Fig. 1). All three genotypes ( ++ , B + and BB) were observed in the Small-tailed Han sheep, Wadi sheep and Tan sheep, while the Hu sheep lacked the ++ genotype. The *FecB* mutation changes glutamine to arginine in sheep. In this study, the B allele and BB genotype were predominant in the Hu sheep and Small-tailed Han sheep. In contrast, the + allele and ++ genotype were predominant in the Tan sheep (Table 2). Genotype distributions were significantly different between the four sheep breeds (Table 3).

**Table 2:** Allele and genotype frequencies of the SNPs of the three fecundity genes in the four sheep breeds.

Gene	Locus	Breed	Hu	Small-	Wadi	Tan	
			sheep	tailed	sheep	sheep	
				Han sheep			
*BMP15*	c.T755C	Number	172	180	160	164	
	(S1)	Genotype frequency	*TT*	0.70(120)	0.75(136)	0.45(72)	0.75(124)
			*TC*	0.14(24)	0.16(28)	0.10(16)	0.15(24)
			*CC*	0.16(28)	0.09(16)	0.45(72)	0.10(16)
		Allele frequency	T	0.77	0.83	0.50	0.83
			C	0.23	0.17	0.50	0.17
*BMPR1B*	c.A746G	Number	172	180	160	164	
	(*FecB*)	Genotype frequency	++	0(0)	0.07(12)	0.22(36)	0.90(148)
			B+	0.19(32)	0.33(60)	0.38(60)	0.05(8)
			*BB*	0.81(140)	0.60(108)	0.40(64)	0.05(8)
		Allele frequency	+	0.09	0.23	0.41	0.93
			B	0.91	0.77	0.59	0.07
*GDF9*	c.G260A	Number	172	180	160	164	
	(G1)	Genotype frequency	*GG*	0.91(156)	0.91(164)	0.90(144)	0.58(96)
			*GA*	0.02(4)	0.09(16)	0.10(16)	0.37(60)
			*AA*	0.07(12)	0 (0)	0(0)	0.05(8)
		Allele frequency	G	0.92	0.96	0.95	0.77
			A	0.08	0.04	0.05	0.23
	c.G477A	Number	172	180	160	164	
	(G3)	Genotype frequency	*GG*	0.44(76)	0.52(92)	0.45(72)	0.32(52)
			*GA*	0.33(56)	0.24(44)	0.43(68)	0.49(80)
			*AA*	0.23(40)	0.24(44)	0.12(20)	0.19(32)
		Allele frequency	G	0.60	0.63	0.66	0.56
			A	0.40	0.37	0.34	0.44
	c.G721A	Number	172	180	160	164	
	(G4)	Genotype frequency	*GG*	0.93(160)	0.84(152)	0.92(148)	0.56(92)
			*GA*	0.07(12)	0.16(28)	0.08(12)	0.22(36)
			*AA*	0 (0)	0 (0)	0(0)	0.22(36)
		Allele frequency	G	0.97	0.92	0.96	0.67
			A	0.03	0.08	0.04	0.33
	c.A1515G	Number	172	180	160	164	
	(S2)	Genotype frequency	*AA*	0.86(148)	0.80(144)	0.80(128)	0.66(108)
			*GA*	0.14(24)	0.13(24)	0.20(32)	0.34(56)
			*GG*	0 (0)	0.07(12)	0(0)	0.27(0)
		Allele frequency	G	0.07	0.13	0.10	0.17
			A	0.93	0.87	0.90	0.83

**Table 3:** Test of the difference in the FecB/G1/G3 loci (above-diagonal) and S1/G4/S2 loci (below-diagonal) genotype distributions of the three fecundity genes in the four sheep breeds.

Breed	Hu	Small-tailed	Wadi	Tan
	sheep	Han sheep	sheep	sheep
Hu sheep		6.1 ${}^{*}$ /4.80	35.1 ${}^{***}$ /4.82	70.0 ${}^{***}$ /16.0 ${}^{***}$
		/0.74	/1.89	/2.36
Small-tailed	1.1/1.60		20.7 ${}^{***}$ /0.03	60.3 ${}^{***}$ /12.7 ${}^{**}$
Han sheep	/2.97		/3.86	/5.69
Wadi	8.14 ${}^{*}$ /0.01	14.4 ${}^{***}$ /1.32		17.3 ${}^{***}$ /10.8 ${}^{**}$
sheep	/0.54	/3.23		/1.73
Tan	0.79/16.5 ${}^{***}$	0.03/12.8 ${}^{**}$	12.7 ${}^{**}$ /15.2 ${}^{***}$	
sheep	/4.72	/7.32 ${}^{*}$	/2.05	

${}^{*}$ P<0.05 ; ${}^{**}$ P<0.01 ; ${}^{***}$ P<0.001 .

### 4.2 Genotyping of *BMP15* mutations

**Figure 2:**
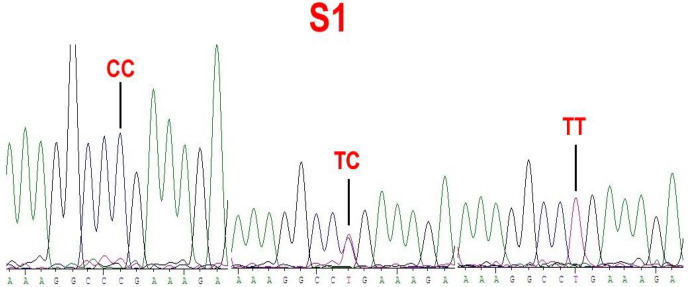
Sequence of genotypes CC, TC and TT at the 755 loci in the CDS region of sheep *BMP15*.

No mutations (*FecX* ${}^{I}$ , *FecX* ${}^{H}$ , *FecX* ${}^{B}$ , *FecX* ${}^{G}$ , *FecX* ${}^{L}$ and *FecX* ${}^{R}$ ) of the *BMP15* were detected in the four sheep breeds. However, a novel point mutation (T755C) (named S1) in the sheep *BMP15* was observed in the four sheep breeds, causing the substitution of a large nonpolar group for a small nonpolar group (Leu252Pro). The S1 mutation had three genotypes, CC, TC and TT (Fig. 2), and the T allele and TT genotype were predominant in the four sheep breeds (Table 2). There were significant differences in the genotype distribution between Wadi sheep and Small-tailed Han sheep ( P<0.001 ), Tan sheep ( P<0.01 ) and Hu sheep ( P<0.05 ), respectively. There was no difference in the genotype distribution among the other three sheep breeds (Table 3).

### 4.3 Genotyping of *GDF9* mutation

**Figure 3:**
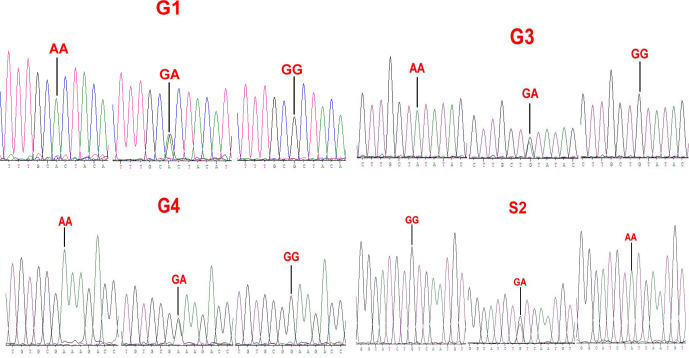
Sequence of genotypes of the G1, G3, G4 and S2 loci in the CDS region of sheep *GDF9*.

Sequence alignment results showed that four nucleotide mutations were found in the sheep *GDF9* gene (Fig. 3), in which a point mutation (G260A) was observed in exon 1 and three SNPs (G477A, G721A and A1515G) were identified in exon 2. Of these four mutations, G260A, G477A and G721A were known as mutations G1, G3 and G4, respectively, which were reported by Hanrahan et al. (2004), and a new point mutation (A1515G) (named S2) was detected in the four sheep breeds. The G3 and S2 mutations were nucleotide changes that did not cause an altered amino acid. G1 and G4 led to amino acid changes, Arg-His and Glu-Lys, respectively. All four mutations gave rise to three genotypes. Small-tailed Han and Wadi sheep lacked AA at the G1 locus. We only detected GG at the S2 locus in Small-tailed Han sheep and AA at the G4 locus in Tan sheep (Table 2).

### 4.4 The haplotype and diplotype analysis of *GDF9*

Interestingly, at the G1 and G4 loci, there were significant differences in the genotype distribution between Tan sheep (low prolificacy) and the other three sheep breeds (high prolificacy). There was no difference in the genotype distribution among the other three sheep breeds at the G1 and G4 loci. For the G3 and S2 loci, except for Tan sheep and the Small-tailed Han sheep ( P<0.05 ) at S2, there was no significant difference ( P>0.05 ) in the genotype distribution among the four sheep breeds (Table 3).

**Figure 4:**
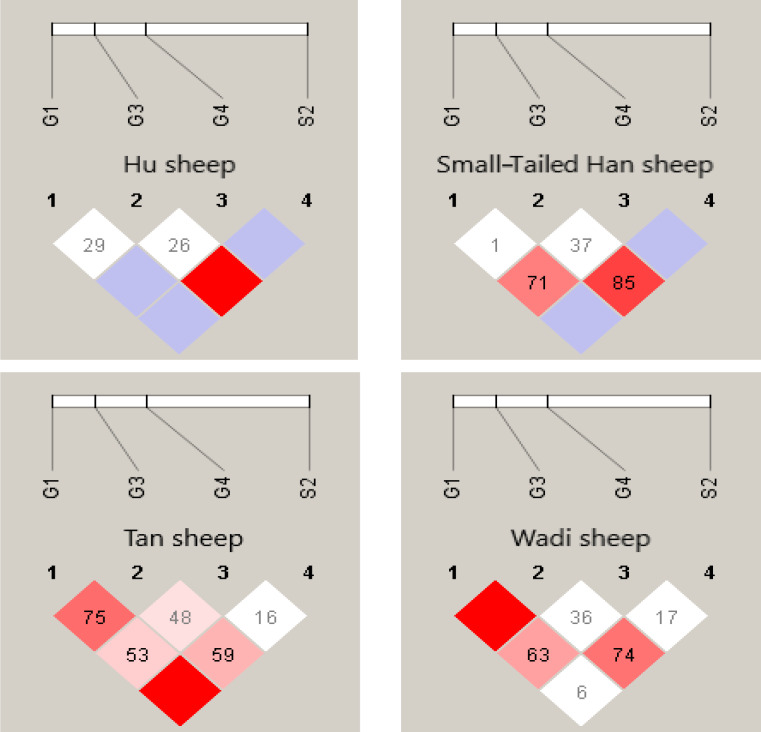
Linkage disequilibrium analyses in the G1, G3, G4 and S2 loci in sheep *GDF9*.

There were 10 haplotypes (Table 4) and 21 diplotypes (Table 5; this was listed when the frequency was larger than 1 %) in the four sheep breeds. H4 was the preponderant haplotype in the three high-prolificacy sheep breeds, while H1 was for Tan sheep. H1 and H4 were the predominant diplotypes in all four sheep breeds. The four sheep breeds were all in linkage equilibrium, and their $r^{2}$ was less than 0.8. The results are shown in Table 6 and Fig. 4.

**Table 4:** Haplotype frequencies of *GDF9* in the four sheep breeds.

Haplotype		Hu	Small-tailed	Wadi	Tan
		sheep	Han sheep	sheep	sheep
H1	GGGG	0.279(96)	0.311(112)	0.288(92)	0.244(80)
H2	GGGA	0.105(36)	0.056(20)	0.125(40)	0.073(24)
H3	GGAG	0.011(4)	0.033(12)	0.025(8)	0.037(12)
H4	GGAA	0.523(180)	0.478(172)	0.475(152)	0.219(72)
H5	GAGG		0.011(4)		
H6	GAGA		0.033(12)	0.025(8)	0.085(28)
H7	GAAA	0.047(16)	0.078(28)	0.050(16)	0.159(52)
H8	AAGG				
H9	AAAA	0.035(12)			0.134(44)
H10	GAAG			0.012(4)	0.049(16)

The numbers of haplotypes are in the parentheses.

**Table 5:** Diplotype frequencies of *GDF9* in the four sheep breeds.

Diplotype	Hu	Small-tailed	Wadi	Tan
	sheep	Han sheep	sheep	sheep
H1/H3		0.022(4)	0.025(4)	0.049(8)
H1/H4	0.488(84)	0.556(100)	0.525(84)	0.367(60)
H1/H7	0.047(8)	0.045(8)	0.025(4)	0.024(4)
H1/H9	0.023(4)			0.098(16)
H2/H4	0.186(32)	0.111(20)	0.225(36)	0.049(8)
H2/H7	0.023(4)		0.025(4)	0.073(12)
H2/H9				0.049(8)
H3/H4	0.023(4)	0.045(8)	0.025(4)	
H4/H4	0.140(24)	0.088(16)	0.075(12)	
H4/H6		0.045(8)	0.025(4)	
H4/H7	0.023(4)	0.022(4)		
H4/H9	0.047(8)			
H5/H7		0.022(4)		
H6/H7		0.022(4)	0.025(4)	0.098(16)
H6/H9				0.024(4)
H6/H10				0.024(4)
H7/H7		0.022(4)		0.024(4)
H7/H9			0.025(4)	0.024(4)
H7/H10				0.049(8)
H9/H9				0.024(4)
H9/H10				0.024(4)

The numbers of diplotypes are in parentheses.

**Table 6:** Linkage disequilibrium analyses in the G1, G3, G4 and S2 loci in sheep *GDF9*.

Breed	L1	L2	$D^{\prime }$	$r^{2}$
Hu sheep	G1	G3	0.291	0.011
	G1	G4	1	0.003
	G1	S2	1	0.007
	G3	G4	0.261	0.004
	G3	S2	1	0.115
	G4	S2	1	0.003
Tan sheep	G1	G3	0.756	0.22
	G1	G4	0.537	0.177
	G1	S2	1	0.062
	G3	G4	0.488	0.15
	G3	S2	0.595	0.093
	G4	S2	0.166	0.003
Wadi sheep	G1	G3	1	0.103
	G1	G4	0.639	0.303
	G1	S2	0.067	0.002
	G3	G4	0.365	0.01
	G3	S2	0.74	0.119
	G4	S2	0.174	0.011
Small-tailed Han sheep	G1	G3	0.018	0
	G1	G4	0.715	0.282
	G1	S2	1	0.007
	G3	G4	0.374	0.02
	G3	S2	0.853	0.193
	G4	S2	1	0.013

When $D^{\prime }=0$ and $r^{2}=0$ , they are in complete linkage equilibrium. When $D^{\prime }=1$ and $r^{2}=1$ , they are in complete linkage disequilibrium.

## 5 Discussion

In sheep, the *BMPR1B*, *BMP15* and *GDF9* genes were found to be closely associated with a genetic basis for prolificacy (Lv et al., 2022; Ji et al., 2023; Zhu et al., 2023). Abdoli et al. (2013) detected polymorphisms of *GDF9* exon 1 and *BMPR1B* exon 8 in Mehraban ewes. The results showed that two SNPs were detected in both genes, and *BMPR1B* exon 8 genotypes appear to have significant effects on reproduction traits, while the *GDF9* genotypes did not have this role. Furthermore, ewes with mutations in both *BMP15* and *BMPR1B* had greater litter sizes than those with either mutation separately, and the effect of the *BMPR1B* mutation was greater than that of the *BMP15* mutation on litter size in sheep (Davis et al., 1999; Davis, 2005; Chu et al., 2007a). To further investigate the type and frequency of mutations in these three genes in Chinese indigenous breeds, we examined the available SNP loci to complement the polymorphism studies of these three candidate genes associated with prolificacy traits in sheep.

*BMPR1B* (*FecB*) was a dominant, autosomal gene that had an additive effect on the ovulation rate and litter size (Davis, 2004). The *FecB* locus is mapped to sheep chromosome 6, corresponding to human chromosome 4 that contains the *BMPR1B*. For the *FecB* mutation, a single A–G substitution at nucleotide position 746 gave rise to an arginine replacing a glutamine (Wilson et al., 2001). Many previous findings identified that the *FecB* was a major gene for high-prolificacy traits in sheep. The *FecB* mutation was initially found in Booroola sheep (Mulsant et al., 2001; Souza et al., 2001; Wilson et al., 2001). Subsequent studies showed that the *FecB* mutation was present in 14 sheep breeds, and half of them were native breeds of China (Chu et al., 2007b; Mahdavi et al., 2014). However, there were some sheep breeds with no *FecB* mutation, including the Chinese Merino, Tan, Dorset and another 24 sheep breeds (Zuo et al., 2013; Jamshidi et al., 2013). One copy of *FecB* increased the ovulation rate by 1.3–1.6 and two copies by 2.7–3.0; the litter size is increased by 0.9–1.2 in ewes carrying a single copy and 1.1–1.7 in ewes with two copies (Piper and Bindon, 1982; Bindon, 1984; Piper et al., 1985). In Hu sheep, Small-tailed Han sheep, Wadi sheep and Altay sheep, ewes with genotype BB or B + had 1.17 ( P<0.001 ) or 1.02 ( P<0.001 ) lambs more than those with genotype ++ (Chu et al., 2011; Li et al., 2012). In this study, the *FecB* mutation was detected in the four (high- and low-prolificacy) sheep breeds, and the frequency of B allele was significantly different in the high-prolificacy breeds than that in the low-prolificacy breed. This result agreed with the previous studies on Chinese sheep breeds, whose fecundities were similar to those reported in Booroola sheep (Reader et al., 2012).

Another important prolificacy-related gene is *BMP15*, which is located on the X chromosome, and five mutations and one deletion are known in sheep breeds. These mutations comprise premature stop codons *FecX* ${}^{H}$ and *FecX* ${}^{G}$ , nonsynonymous amino acid substitution *FecX* ${}^{I}$ , *FecX* ${}^{B}$ and *FecX* ${}^{L}$ and a 17 bp deletion of the reading frame *FecX* ${}^{R}$ (Galloway et al., 2000; Hanrahan et al., 2004; Bodin et al., 2007; Martinez-Royo et al., 2008; Monteagudo et al., 2009). The above six mutations in *BMP15* were associated with both increased ovulation rates and litter sizes in heterozygous carriers as well as sterility in homozygous carriers (Hanrahan et al., 2004). Studies suggested that heterozygous ewes for these mutations usually increased ovulation rates (without drastic changes in gonadotrophin secretion), while performances of homozygous ewes were early streak ovaries, folliculogenesis arrest and ovarian dysgenesis. The heterozygous deletions reduced *BMP15* concentration, increased granulosa cell follicle-stimulating hormone receptor (FSHR) concentration, elevated estrogen secretion and activated the production of stem cell factors (SCFs) (Abir and Fisch, 2011). Without bone morphogenetic protein 15, oocytes will degenerate in the absence of granulosa cell proliferation (Galloway et al., 2000). These findings highlighted the importance of *BMP15* in regulating ovulation rates. In our study, no polymorphism was detected in any of these six mutations of sheep *BMP15*. However, the novel mutation S1 (T755C) was observed in the four sheep breeds, causing the substitution of a large nonpolar group for a small nonpolar group (Leu252Pro). This mutation might be important in causing differences in lambing numbers in native Chinese sheep breeds.

In sheep, *GDF9* has been mapped to chromosome 5 and has encoded a prepropeptide of 453 amino acid residues, which could generate an active mature peptide with 135 amino acids (Bodensteiner et al., 1999). *GDF9* plays a crucial role as a growth and differentiation factor secreted by oocytes during early folliculogenesis in female reproduction (Elvin et al., 1999). Many findings indicated that *GDF9* could regulate several essential granulosa cell enzymes which participated in cumulus expansion and maintenance of an optimal oocyte microenvironment through oocyte–somatic cell interaction and synergistic action (Yan et al., 2001; McNatty et al., 2005b). Several mutations have been observed in sheep *GDF9*. Li et al. (2003) detected a point mutation (A152G) of the *GDF9* gene in Hu, Dorset and Suffolk sheep that gave rise to an amino acid change (Asn51Asp). Eight DNA variants (G1–G8) in the entire *GDF9* coding region of Cambridge and Belclare sheep were observed (Hanrahan et al., 2004). *FecTT* mutation (A1279C) was found in the *GDF9* of Icelandic Thoka sheep, leading to a change in serine (neutral) to arginine (basic) at the 109th residue in a mature *GDF9* peptide (Nicol et al., 2009). *FecG* ${}^{E}$ mutation (T1034G) in the *GDF9* gene of Brazilian Santa Ines sheep has been detected (Silva et al., 2011). Chu et al. (2011) found one novel single-nucleotide mutation (G729T) in exon 2 of *GDF9*, which led to an amino acid change (Gln243His). For all these mutations, heterozygous ewes with the G1, G8 or *FecTT* mutations have shown an increase in ovulation rate and fecundity. In contrast, the *FecG* ${}^{E}$ mutation in *GDF9* led to an increased ovulation rate in homozygous ewes (Hanrahan et al., 2004; Nicol et al., 2009; Silva et al., 2011). In this study, four mutations (G1 [G260A], G3 [G477A], G4 [G721A] and S2 [A1515G]) have been observed in the four sheep breeds, of which the S2 mutation did not result in an altered amino acid. Our results were similar to those of previous studies. In particular, we first detected the G1, G3 and G4 mutations simultaneously in Wadi sheep, which are a high-prolificacy local sheep breed in China. Haplotype analysis revealed that H4 (GGAA) was the preponderant haplotype in the three high-prolificacy breeds, but H1 (GGGG) was in Tan sheep (Table 4). H1 and H4 were the predominant diplotypes in all the sheep breeds (Table 5).

## 6 Conclusion

In conclusion, we explored polymorphisms in three critical fecundity genes in the Hu sheep, Small-tailed Han sheep, Wadi sheep and Tan sheep breeds. For *BMPR1B*, the *FecB *mutation was present in all four sheep breeds. The frequency of B allele in the high-prolificacy breeds was significantly higher than that in the low-prolificacy breed ( P<0.001 ). For *BMP15*, a novel mutation (c.T755C) was found, while six known mutations (*FecX* ${}^{I}$ , *FecX* ${}^{H}$ , *FecX* ${}^{B}$ , *FecX* ${}^{G}$ , *FecX* ${}^{L}$ and *FecX* ${}^{R}$ ) were not detected in the four sheep breeds. For *GDF9*, the three known mutations (*FecG* ${}^{H}$ (G8), *FecG* ${}^{E}$ and *FecTT*) were not detected, and three known loci (G1, G3 and G4) as well as a new mutation, c.A1515G (named S2), were found in the four sheep breeds. The predominant alleles and genotypes are different between the high- and low-prolificacy breeds. At the G1 and G4 loci, there were significant differences in the genotype distribution between Tan sheep (low prolificacy) and the other three sheep breeds (high prolificacy), and there was no difference in the genotype distribution between the other three sheep breeds. Haplotype analysis of the four polymorphic loci in *GDF9* indicated that H4 (GGAA) and H1 (GGGG) were the preponderant haplotypes in the high-prolificacy sheep breeds and the low-prolificacy breed, respectively. These results preliminarily showed that the *BMPR1B* and *GDF9* might be major genes influencing the prolificacy of Chinese sheep breeds.

## Data Availability

No data sets were used in this article.
